# Molecular Interactions Between Vascular Smooth Muscle Cells and Macrophages in Atherosclerosis

**DOI:** 10.3389/fcvm.2021.737934

**Published:** 2021-10-15

**Authors:** Jahnic Beck-Joseph, Maryam Tabrizian, Stephanie Lehoux

**Affiliations:** ^1^Biomat'X Research Laboratories, Department of Biomedical Engineering, McGill University, Montreal, QC, Canada; ^2^Department of Medicine, Lady Davis Institute, McGill University, Montreal, QC, Canada

**Keywords:** atherosclerosis, coronary artery disease, inflammation, cell crosstalk, smooth muscle cells, monocytes/macrophages, immunity

## Abstract

Atherosclerosis is the largest contributor toward life-threatening cardiovascular events. Cellular activity and cholesterol accumulation lead to vascular remodeling and the formation of fatty plaques. Complications arise from blood clots, forming at sites of plaque development, which may detach and result in thrombotic occlusions. Vascular smooth muscle cells and macrophages play dominant roles in atherosclerosis. A firm understanding of how these cells influence and modulate each other is pivotal for a better understanding of the disease and the development of novel therapeutics. Recent studies have investigated molecular interactions between both cell types and their impact on disease progression. Here we aim to review the current knowledge. Intercellular communications through soluble factors, physical contact, and extracellular vesicles are discussed. We also present relevant background on scientific methods used to study the disease, the general pathophysiology and intracellular factors involved in phenotypic modulation of vascular smooth muscle cells. We conclude this review with a discussion of the current state, shortcomings and potential future directions of the field.

## Introduction

Cardiovascular disease is the leading cause of death worldwide with atherosclerosis being the largest contributor toward cardiovascular events such as myocardial infarctions and strokes (1, 2). The disease is characterized by systemic inflammation and a buildup of fatty plaques in the arterial vessel wall (3). Medical complications arise through the restriction of blood flow due to lumen encroachment by the plaque or thrombosis occurring at sites of plaque rupture or erosion (4). Atherosclerosis develops over many years and progresses through a complex interplay between vascular cells, infiltrating leukocytes, endothelial shear stresses, and systemic factors such as liver-induced impairment of fibrinolysis or signaling from adipose tissue (4–6).

Elevated levels of cholesterol-associated apolipoprotein (apo) B are required for the development of atherosclerosis in humans and animal models (7). Intimal retention of these lipoproteins is facilitated by the production of proteoglycans from vascular smooth muscle cells (VSMCs). In response to sub-endothelial accumulation and oxidation of low-density lipoprotein (LDL), circulating leukocytes infiltrate into the arterial vessel wall. This critical event typically initiates the onset of the disease and the formation of early atherosclerotic lesions. Inside the vessel, monocytes differentiate into macrophages, engulf residential LDL, and adopt a pro-inflammatory phenotype (3, 8). Many diverse cell-types play significant roles in disease progression. However, in numbers, macrophages and VSMCs dominate the landscape. Both cell types retain the capacity to engulf modified LDL and gradually transform into lipid-laden foam cells that amplify atherogenesis and aggravate the disease (4).

Hence, VSMCs and macrophages undergo remarkably diverse phenotypic transitions and their derived cell types play dominant roles in developing plaques. Furthermore, the fate of these cells is pivotal for disease progression and outcome (4, 9–11). Interactions between both cell types and their influence on the disease are still not well-understood. A better comprehension of the molecular changes they undergo might lead to new insights on how to prevent or revert disease progression; this may be particularly true at early stages where lesional complexity is low and cells might be amenable to reset toward a more reparative phenotype.

### Experimental Approaches

Mouse models with genetic deletions such as ApoE–/– or LDL receptor–/–, which prime animals toward hypercholesterolemia and atherosclerosis, have been used extensively to explore the disease in live organisms. Additional gain- or loss-of-function mutations have been applied to investigate the impact of specific gene products or cell types on lesion progression. When applied to bone marrow transplants, they are useful for exploring the function of specific genes in hematopoietic vs. non-hematopoietic cells (12). Additionally, specific myeloid or lymphoid cells can be targeted at a chosen time using tamoxifen-inducible CreERT2 recombinase under the control of a cell-specific promoter such as LysM (macrophages and neutrophils), F4/80 (tissue macrophages), or CX3CR1 (certain monocytes and macrophages) (13). Alternatively, the loxP-Cre system can induce tissue-specific, cell-specific, or timed genetic modifications. Cre-recombinase activity results in targeted genetic excision around sequences sandwiched (floxed) between two loxP sites. Expression of the enzyme can be placed under a tissue/cell-specific or chemically inducible (e.g., tamoxifen) promoter, which allows for a wide range of spatial and temporal control (12, 14). The degree of exploratory freedom that genetic manipulations in live animals offer has played an important role in studying atherosclerosis. However, inter-species differences can limit the translatability of animal models to humans. This limitation is offset by the fact that human studies are expensive and restricted to post-intervention assessments without the power of *in vivo* genetic interventions (15).

The intricacy of atherosclerotic lesions complicates the interpretation of scientific observations. High-resolution techniques such as single-cell RNA-Seq in combination with computational data analysis have great potential to unravel this complexity. However, large amounts of pure cells are required, which can be challenging for small model organisms such as mice. The technology is further limited by technical drawbacks. Errors and biases are introduced at various steps such as during RNA amplification or cell lysis. Most notably, only 10-40% of all transcripts in a particular cell are captured and converted into cDNA (16). Additionally, inferring the function of a particular cell in its environment is currently challenging since metadata on cellular position is generally not available. This is a considerable drawback given that cells are expected to behave vastly differently depending on their position within the plaque and their immediate microenvironment (17). Lastly, the high price for sequencing on the single-cell level currently hampers widespread usage and high-throughput experimentation.

*In vitro* cultures of animal and human cells have been extensively used to abstract from the complex nature of the disease and focus on simpler, isolated aspects. Historically, three co-culture types have been used to address cell-cell interactions: (1) indirect contact with physically separated cell types, (2) direct cell contact, and (3) 3D scaffolds attempting to model features of the vasculature (18). While a lower level of complexity can be helpful, oversimplification of *in vivo* conditions can also be problematic. Factors relevant to the studied interaction might be missing. VSMCs in culture are known to undergo phenotypic switching with reduced contractility, enhanced proliferation, and susceptibility toward apoptosis (19–21). Cells with corrupted phenotypes may produce misleading results that are not transferable to live animals or humans.

## Pathophysiology

Pathological intimal thickenings (PITs) are believed to characterize the onset of atherosclerosis and develop from diffuse intimal thickenings (DITs) of the arterial vessel wall. DITs are characterized by their high proteoglycan content which facilitates the retention of apolipoproteins. Through continuous lipid retention, monocyte recruitment and foam-cell formation, DITs may progress toward sites of chronic inflammation with enhanced production of inflammatory mediators such as tumor necrosis factor α (TNF-α), interleukin-1 (IL-1), and macrophage colony-stimulating factor (M-CSF) (4, 22). Additional molecular processes occur that favor disease progression, such as accumulation of reactive oxygen species (ROS) and increased nitric oxide (NO) production (23, 24). Elevated levels of apolipoprotein B (apo B) containing lipoproteins such as LDL and VLDL are a prerequisite for atherosclerotic plaque development, independent of additional risk factors (7). Other critical events include enhanced endothelial permeability and increased monocyte recruitment from the circulation (23–25).

Atherogenic processes are often interconnected and may substitute or influence each other. For example, the transcription factor NF-κB is both redox-sensitive and a well-established master regulator of inflammation (26). Oxidative stress may, therefore, lead to inflammation and hence activation of endothelial cells (ECs). Both permeability and upregulation of surface binding proteins are enhanced in activated ECs and may facilitate monocyte infiltration (24, 27). Release of chemokines largely regulates the attraction of monocytes to the endothelial layer where binding occurs *via* surface proteins such as vascular cell adhesion molecule 1 (VCAM-1) and intercellular adhesion molecule 1 (ICAM-1). Infiltrating monocytes may differentiate into macrophages and further drive atherogenic processes including EC activation and inflammation.

### Early Lesions

Within the intima, lipids are modified by resident oxygen radicals and enzymes, which precedes macrophage recruitment. Particularly, the production and retention of oxidized low-density lipoproteins (oxLDL) promote the differentiation of VSMCs into foam cells. Hence, early DITs contain mainly dedifferentiated VSMCs. These in turn synthesize proteoglycans that facilitate the retention of LDL. Proteoglycans play a critical role in early lesion formation but are not necessary for atherosclerosis to proceed (7, 28). Experiments showed no difference in advanced plaques from mice with proteoglycan binding deficient LDL and normal LDL (28). LDL engulfment also transforms intimal macrophages into lipid-laden foam cells, which are a hallmark of early pathogenesis (26, 29, 30). Once fully differentiated, foam cells are largely immobilized, sustain inflammation, and exhibit increased rates of apoptosis (23). Progression toward pathological intimal thickenings (PITs) is promoted through enhanced subendothelial LDL retention, LDL modification, inflammation, apoptosis, and phenotypic switching of VSMCs (4).

### Advanced Lesions

The majority of life-threatening cardiovascular events arise from the rupture or erosion of advanced atherosclerotic plaques. Forming blood clots around the damaged vascular site may detach and form thrombotic occlusions further downstream in the vasculature. Eroded plaques tend to have an intact fibrous cap rich in ECM components and occur because of endothelial cell desquamation (31). In contrast to ruptured plaques, eroded lesions typically have lower lipid levels, fewer inflammatory cells such as macrophages and lymphocytes, and contain more VSMCs. The forming thrombus is also different between the two types. Thrombi formed during plaque erosion contain mainly platelets, whereas those from plaque rupture are rich in fibrin (31, 32).

Advanced atherosclerotic plaques are generally characterized by regions of enhanced cell death, calcification, necrotic core formation, and defective efferocytosis, which is the process by which apoptotic debris are cleared through phagocytic activity (4, 5, 33). VSMCs take on diverse roles as plaques evolve. Synthetic VSMCs near the endothelium produce a collagen-rich fibrous cap, which protects the plaque from rupture. Consequently, low VSMC counts and enhanced inflammation have long been associated with plaque vulnerability (5, 22, 33, 34). The presence of macrophages at the fibrous cap may further compromise plaque stability by initiating VSMC apoptosis, contributing toward inflammation, and secreting MMPs (22, 33–35). In addition, plaque VSMCs can differentiate into osteochondrogenic-like cells, releasing minerals and bone-generating factors (4). The impact of calcification on plaque stability seems largely a function of the pattern, location, and size of calcium aggregates. Spotty microcalcification with the formation of nodules near the fibrous cap appears to enhance vulnerability, whereas sheet-like macrocalcifications are associated with more stable plaques (36, 37). However, macrocalcification of the medial layer also confers arterial stiffness which may lead to systemic cardiovascular complications such as hypertension that may ultimately aggravate or initiate atherosclerosis (38). Chronic VSMC apoptosis in mice has also been linked to enhanced intimal calcification (39). In VSMC cultures, caspase targeted inhibition of apoptosis led to a 40% reduction of calcified nodules, while stimulation of death receptor Fas led to a 10-fold increase (40). These findings suggest VSMC death to be a significant factor in vascular calcification. Additionally, it was shown that apoptotic bodies, released during apoptosis, may serve as nucleation points for the formation of concentrated spotty calcium residues (40, 41). Mineral deposits, in turn, have been shown to induce cell death in VSMCs (21, 42). This may constitute a positive feedback loop *in vivo* where VSMC apoptosis and intimal calcification amplify each other. Thus, defective efferocytosis in combination with increased cellular death may be pivotal for plaque calcification. Furthermore, defective clearance leads to the aggregation of dead cell debris and lipids to form a growing necrotic core that weakens plaque integrity. Uncleared apoptotic cells may also undergo secondary necrosis and release potent proatherogenic cytokines that enhance inflammation and aggravate the disease (4, 19).

The use of single-cell sequencing in human and mice studies has recently identified lesional macrophage subsets that exceed the canonical surface marker-based paradigm. Furthermore, commonly used markers were expressed across distinct populations, suggesting that macrophage subtypes and functionalities may not be as clearly delimited as previously assumed. Interestingly, phenotypic diversity of lesional macrophages appeared to increase over time, which may be a direct consequence of the evolving plaque complexity (10, 43, 44). Given the high degree of macrophage diversity and their importance in atherosclerosis, cues that influence the balance of macrophage differentiation may have a critical role in disease progression and outcome.

### Sex Differences

Significant age and sex differences exist in the incidence and constitution of plaques. Prior to menopause, women are relatively protected from cardiovascular disease with smaller necrotic core volumes and generally more stable plaques, which suggests a protective role for estrogen (45, 46). Estrogen in mice leads to a reduction of macrophage recruitment through two distinct mechanisms. Estrogen reduces endothelial expression of ICAM-1 and VCAM-1, which are critical for leukocyte binding, and it downregulates the synthesis of monocyte attracting chemokines such as CCL2. There is also evidence that estrogen regulates inflammation. Young women exhibit peak annexin A1 expression when serum estrogen levels are at their highest. Annexin A1 is an anti-inflammatory that plays a central role in the phagocytic removal of apoptotic leukocytes (efferocytosis). Furthermore, estrogen also acts on macrophages *via* estrogen receptors α and β. Receptor activation promotes macrophage polarization to an anti-inflammatory phenotype and the release of inflammation resolving factors such as IL-10 and TGF-β (46). Interestingly, a recent investigation of gene regulatory networks (GRNs) discovered differences between the sexes in the expression of key genes involved in atherosclerosis. Single-cell RNA sequencing of mice plaques suggested that phenotypically modulated VSMCs were responsible for a majority of the detected GRN drivers in females. Consequently, sex differences in the regulation of VSMC phenotype modulation may also play a significant role in the observed differences in cardiovascular disease (47).

## VSMC Phenotypic Modulation

VSMCs in atherosclerosis may accumulate in the tunica intima and transition to several distinct cell types. Signals from the micro-environment lead to transcriptional changes that result in phenotypic modulation. During this process, VSMC markers are lost, and genes typical of other cell types are up-regulated (48). Three major sub-populations have been identified. Synthetic VSMCs secrete extracellular matrix (ECM) components that increase LDL retention in early atherosclerotic lesions and stabilize the developing plaque at later stages. The osteochondrogenic-like cell type may enhance plaque vulnerability by excreting mineral deposits. Lastly, VSMCs may transition to a foam cell phenotype with surface markers indistinguishable from macrophages (4, 29).

### Migration and De-differentiation

The majority of intimal VSMCs originate from vascular smooth muscle cells of the tunica media. Medial VSMCs detach and de-differentiate into a synthetic phenotype that produces and modulates extracellular matrix (ECM) components (4). Other cell populations such as myeloid, endothelial, and vascular resident stem cells may contribute to intimal VSMC accumulation by differentiating into an VSMC-like phenotype. Their relative abundance and impact on disease development remain, however, controversial (49–52). De-differentiation of medial cells is typically initiated in response to external cues such as vascular injury or inflammation. Agents known to promote VSMC migration are primarily growth factors and inflammatory cytokines such as angiotensin II (Ang II), vascular endothelial growth factors (VEGF), interleukin-6 (IL-6), and TNF-α but also extracellular matrix components including collagen I, IV, and VIII. Through the activation of receptor tyrosine kinases (RTKs) and G protein-coupled receptors (GPCRs), external signals are relayed and induce the remodeling of the cytoskeleton. Major pathways include the phosphorylation of various kinases such as Rho-, p21-, and mitogen-activated protein kinases. Downstream targets, such as actin-related protein 2/3 and heat shock protein 27, are involved in actin polymerization or participate in generating traction forces such as myosin II (53). Synthesized matrix metalloproteinases, such as MMP-2, -3, and -9 degrade cellular adhesion proteins and enable the mobilization of VSMCs into the intimal layer (54, 55).

MMP expression may influence plaque progression in various ways. Collagenases such as MMP-1, -8, and -13 have been associated with plaque vulnerability and may directly participate in degrading the collagen-rich fibrous cap (56, 57). MMPs have been implicated in a wide range of additional atherogenic processes such as enzymatic modification of LDL (MMP-2/MMP-9) (58), inflammation (MMP-14) (59), cellular proliferation (MMP-9/MMP-14) (59–61), apoptosis (MMP-7/MMP-12) (57), and vascular calcification (MMP-2) (62). Consequently, MMPs can have positive and negative influences on disease progression, depending on the surrounding context of MMP synthesis and which types are expressed.

### Macrophage Marker Expression

Foam cell formation is a hallmark of early atherogenesis. These cells exhibit unbalanced uptake, esterification, and efflux of lipids, leading to an accumulation of cholesterol droplets and a foamy appearance (26). Foam cells were initially believed to originate exclusively from infiltrating monocytes due to the expression of surface markers typical for macrophages. The scavenger receptor Cluster of Differentiation 68 (CD68), for example, is expressed by foam cells and typically found on cells of monocyte lineage. However, recent studies have shown that VSMCs contribute significantly to CD68 positive foam cells in human and mouse atherosclerotic lesions. Through uptake of LDL, VSMCs initiate the transition toward a dysfunctional macrophage-like phenotype. In the process, smooth muscle-markers such as smooth muscle α-actin (SMA) and myosin heavy chain (MYH11) are either down-regulated or lost entirely. In contrast, macrophage-associated markers such as ABCA1, CD68, and LGALS (MAC-2) are up-regulated (9, 63). Studies in live mice have highlighted Kruppel-like factor 4 (KLF4) as a central agent in oxLDL-induced phenotypic switching of VSMCs. Furthermore, loss of KLF4 has been associated with a marked reduction in plaque size and increased fibrous cap thickness without affecting the overall number of lesional VSMCs (55). This suggests that phenotypic VSMC modulation and the relative abundance of different phenotypes have a stronger influence on plaque stability than total intimal VSMC count. Compared to foam cells derived from macrophages, smooth muscle-derived foam cells appear to exhibit lower expression of ABCA1, which may suggest a reduced capacity for cholesterol efflux (9). Therefore, VSMCs may have a higher propensity to transform into foam cells and to resist cholesterol-efflux-dependent plaque regression.

### Calcification

Arterial intimal calcification (AIC) is positively correlated with plaque burden, progression, and vulnerability (36, 64). Fate mapping studies have identified VSMC-derived osteoblast- and chondrocyte-like cells as significant contributors toward AIC in tandem with bone marrow-derived cells (65, 66). In human atherosclerotic plaques, osteogenic VSMCs are associated with lipid-rich, calcified regions. Cholesterol accumulation in VSMC cultures is required for calcification and promotes the expression of factors involved in osteogenic differentiation, such as alkaline phosphatase and bone morphogenic protein (BMP-2) (41, 67). Particularly modified LDLs enhance calcification and phenotypic modulation (30). The master regulator runt-related transcription factor 2 (Runx2) is pivotal for osteochondrogenic differentiation of VSMCs and vascular calcification (38).

Oxidative stress in the form of accumulating ROS and inflammatory cytokines, such as IL-6 and TNF-α, have been linked to increased Runx2 expression and are believed to play essential roles in vascular calcification (38, 68, 69). In a recent study on mice, vascular smooth muscle-specific KO of Runx2 significantly reduced AIC without affecting other pivotal aspects of the disease, such as atherosclerotic lesion formation or macrophage content. Different stages of chondrocyte differentiation were examined by measuring VSMC expression of Sox9 (prechondrogenic fate decision), Col II (early differentiation), and Col X together with MMP13 (maturation). Early markers were unaffected by the Runx2 deficiency, while MMP13 and Col X were significantly inhibited, suggesting that Runx2 is involved in the maturation process rather than the early stages of phenotypic switching (38). Further downstream, expression of the membrane-bound alkaline phosphatase TNAP ultimately enables extracellular phosphate capturing and mineralization (67). Recent evidence shows that the pattern of intimal calcification is relevant to plaque burden and vulnerability (36).

## Molecular Interactions Between VSMCs and Macrophages

**Figure 1:**
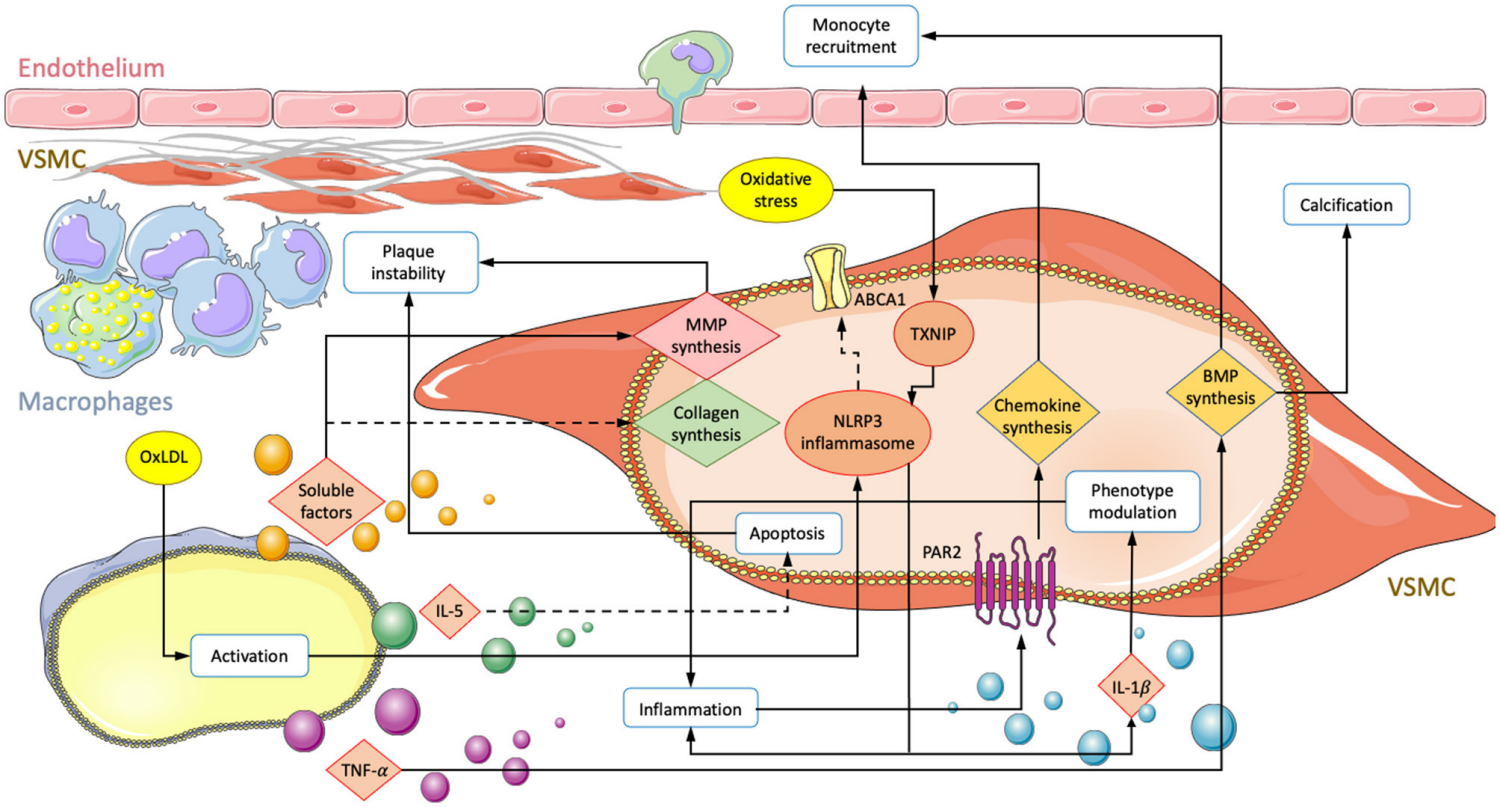
Summary of interactions involving an exchange of soluble factors between macrophages and VSMCs in atherosclerosis. Depiction of a vessel with an endothelial layer on the top and fibrous cap producing VSMCs lying underneath. Macrophage secretion of unestablished soluble factors, TNF-α, IL-5, and oxLDL mediated activation lead to transcriptional changes in VSMC. Solid arrows correspond to enhanced activity and dotted lines represent down regulated processes. Modifications result in increased monocyte recruitment, plaque instability, subendothelial calcification, and inflammation.

Within the atherosclerotic micro-environment, macrophages and VSMC are found in close proximity. Intercellular interactions occur through direct contact or the exchange of soluble factors and are believed to have a significant impact on the disease (Figure 1) (3, 70, 71).

### Exchange of Soluble Factors

#### VSMC to Macrophage Signaling

Macrophage recruitment is an important step in atherosclerotic lesion formation, and although monocyte adherence to endothelial cells is crucial, VSMCs also play a critical role in this process by releasing chemoattractants such as CCL2, CXCL1, and bone morphogenic proteins (BMPs) (72–74). The presence of oxLDL in the vessel wall stimulates the production of chemoattractants in both ECs and VSMCs (75). One study has implicated protease-activated receptor 2 (PAR2) in macrophage recruitment. PAR2 is involved in the inflammatory process and localizes to intimal VSMCs in atherosclerotic lesions (73, 74). Its activation in primary VSMCs of mice was shown to increase the secretion of CCL2 and CXCL1. *In vivo* knockout of PAR2 in vascular cells resulted in reduced expression of CCL2/CXCL1 and lower macrophage content in atherosclerotic lesions. Further, plaques were more stable, with increased smooth muscle ACTA-2 and collagen content and reduced inflammatory factors such as IL-1 and TNF-α (76).

VSMC-expressed BMP-2 and BMP-4 may also participate in the recruitment of macrophages to atherosclerotic lesions. Migration assays on primary mouse cells have shown that both BMP-2 and-4 attract monocytes through activation of BMPRII. The presence of antagonist Gremlin or siRNA significantly reduced migration (77). Furthermore, studies have shown that macrophages enhance BMP-2 expression in VSMCs by releasing proinflammatory cytokines such as TNF-α (78). Bone morphogenic proteins are implicated in a variety of atherogenic processes, including calcification (67), plaque instability (76), inflammation (77), and phenotypic modulation of VSMCs (65, 79). TNF-α has also been shown to increase alkaline phosphatase (ALP) activity and calcification in human VSMC cultures (80, 81). The BMP-TNF-α axis thus constitutes a link between macrophage recruitment and vascular calcification *in vivo*.

In the vessel wall, growth factors are generally considered to induce proliferation and amplification, but they also contribute to macrophage recruitment. In a model of angiotensin II (Ang II)-induced vascular remodeling, activation of the key regulatory transcription factor hypoxia inducible factor 1-alpha (HIF1α) was examined. Cre-recombinase-controlled HIF1α deficiency in VSMCs revealed a central role for macrophage recruitment through CCL7 signaling. Both VSMC HIF1α deficiency and CCL7 neutralization, respectively, suppressed the Ang-II induced recruitment of macrophages and subsequent vascular remodeling in mice. Further, HIF1α depletion disrupted only accumulation of CD206– macrophages consistent with a M1 phenotype. Infiltration of CD206+ macrophages, T-cells, and neutrophils were unaffected, suggesting a specific recruitment of inflammatory macrophages (82).

#### Macrophage to VSMC Signaling

A 2016 study employed transwell co-cultures of human aortic VSMCs and PMA-activated macrophages. Synthesis of Col I and Col III in VSMCs were demonstrated to be significantly reduced, while MMP1/9 expression was increased in both VSMCs and macrophages in response to co-culturing. This may decrease plaque stability from two angles. First, by reducing collagen synthesis and, second, by degrading ECM components of the fibrous cap. Furthermore, both cell types showed elevated expression levels of IL-1β, TLR-2, and VEGF-A, which may further reduce plaque stability through inflammation, neovascularization, and calcification (56, 70, 71, 83).

However, *in vivo* implications might be less straightforward. VEGF-A for example has also been demonstrated to be atheroprotective by promoting endothelial repair during vascular injury (84). Recently, macrophages were shown to attenuate VSMC apoptosis. IL-5 expression was found to be decreased in aortas from patients with acute aortic dissection (AAD) and localized to macrophages (35, 85). In membrane-separated co-cultures with primary mouse cells, macrophage IL-5 overexpression significantly reduced VSMC apoptosis by modulating the death regulators Bax and Bcl-2 (35). Macrophages exhibiting increased IL-5 expression may keep VSMC numbers high and consequently be beneficial for maintaining plaque stability. However, reduced ECM synthesis combined with increased VSMC survival may also lead to the accumulation of pathological phenotypes such as foam or calcifying cells. Such findings underline the importance of understanding the function of cellular subtypes and their roles in different pathological environments.

A recent study demonstrated nucleotide-binding oligomerization domain-like receptor protein 3 (NLRP3) inflammasome activation in VSMCs after co-culture with oxLDL-activated monocytes (34). NLRP has previously been demonstrated to play a significant role in atherosclerosis and was shown to transform VSMCs into a macrophage-like phenotype. NLRP3 induced VSMC proliferation and modulation to a foam cell-like phenotype was dependent on NF-κB activation (86–88). *In vivo*, Apoe–/– mice fed a high cholesterol diet were found to exhibit an increase in VSMCs expressing both CD68 and NLRP3 compared to control mice fed without cholesterol (88). Consequently, macrophages in combination with LDL may promote disease progression and VSMC differentiation to foam cells through NLRP3 inflammasome activation. Foam cell formation from LPS-stimulated primary human VSMCs was shown to depend on NLRP3-induced secretion of high mobility group box-1 protein (HMGB1) and subsequent intracellular lipid accumulation. Loss of function experiments that impaired HMGB1 binding to receptor for advanced glycation end product showed increases in ABCA1 expression and cholesterol accumulation (87). NLRP3 activation in VSMCs has also been shown to lead to maturation and secretion of IL-1β and IL-18 (89). Both cytokines play key roles in atherosclerosis and may participate in modulating VSMCs to a migratory phenotype with enhanced proliferation and expression of inflammatory markers (90–92). Lastly, NLRP has been linked to gasderminD-dependent pyroptosis in VSMCs, which occurs ubiquitously in atherosclerotic lesions and is a form of cell death that impacts atherogenesis, inflammation, and plaque instability (93).

During arterial injury-induced vascular remodeling, Cre-LoxP-directed deletion of dynamin-1-like protein (Drp1) in macrophages significantly suppressed intimal thickening and macrophage infiltration. VSMCs cultured indirectly with macrophages from macrophage-Drp1-KO mice showed reduced proliferation compared to control cells cultured with wild-type macrophages. Additionally, VSMC migration was found to be significantly reduced in media conditioned by macrophage-Drp1-KO rather than normal macrophages. Furthermore, Drp1 knockout and loss-of-function in activated macrophages led to decreases in inflammatory marker expression, including the macrophage attractant CCL2 and platelet-derived growth factor subunit B (PDGF-B); transient overexpression had opposite effects (94). In addition to proliferation and hyperplasia, PDGF-B overexpression in VSMCs has previously been reported to induce the expression of a wide range of monocyte attracting CC-chemokines and inflammatory cytokines. Expression changes were found to be primarily regulated through activation of signal transducer and activator of transcription 1 (STAT1). Increased PDGF-B expression was also shown to dedifferentiate VSMCs into a phenotype consistent with ECM-producing synthetic cells (95). These findings may suggest a positive feedback loop between VSMCs and macrophages that at least in part is regulated by macrophage Drp1 expression. During vascular remodeling, macrophages may initiate VSMC dedifferentiation, migration, proliferation, and expression of monocyte-attracting chemokines. The latter leads to further accumulation of macrophages and the closing of the loop. Additionally, the findings suggest that PDGF-B expression could prime incoming VSMCs toward a reparative phenotype.

### Extracellular Vesicle Signaling

**Figure 2:**
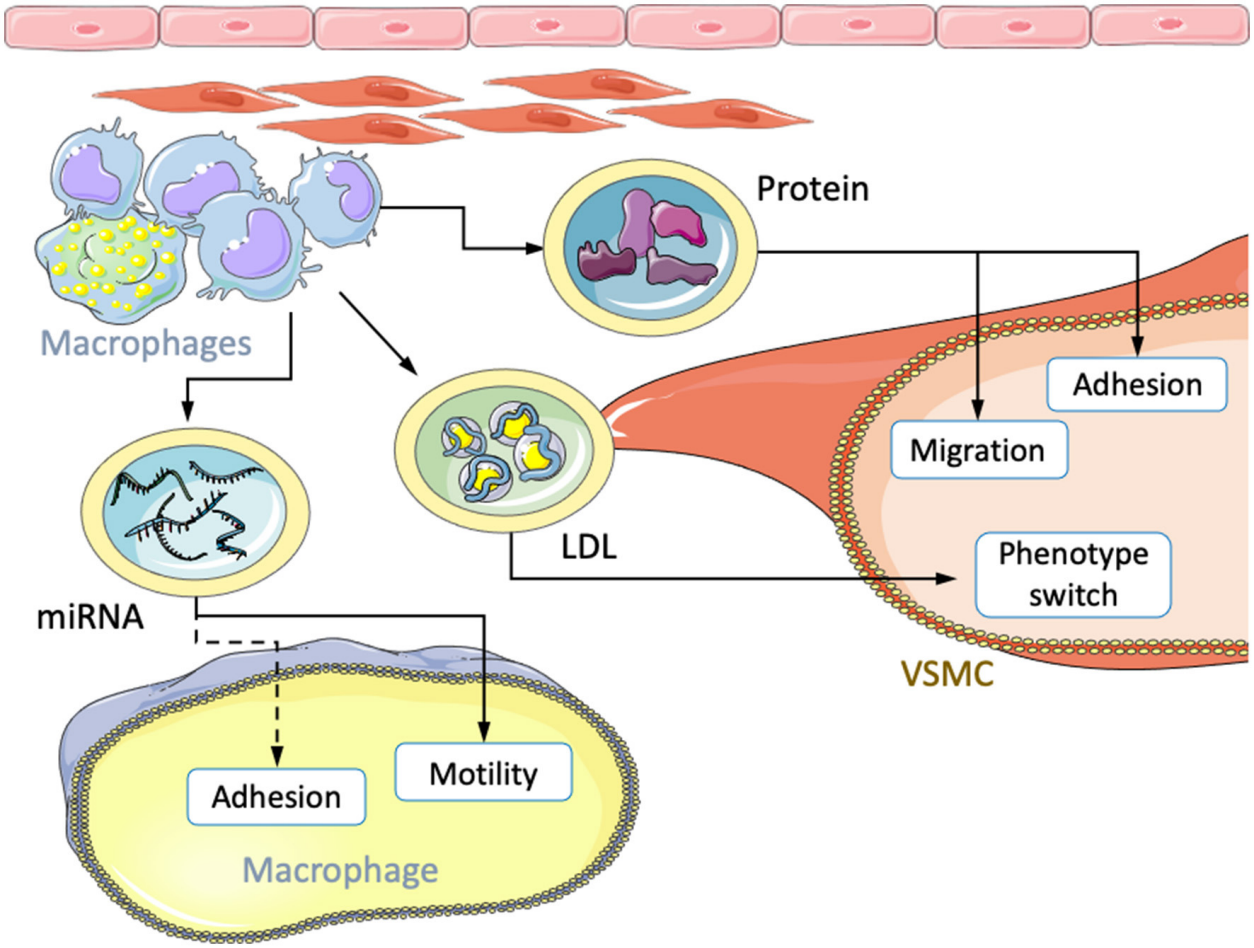
Transfer of atherosclerotic factors *via* extracellular vesicles. Macrophages transfer miRNA, LDL, and protein cocktails to other macrophages and VSMCs. The received content leads to changes in migration, motility, adhesion and phenotypic modulation.

Extracellular vesicles (EVs) are a more recent paradigm of intercellular interactions and involve the transfer of complex protein, genetic, or lipid cocktails *via* endocytosis (Figure 2). The conferred content modulates gene expression in the recipient cells leading to physiological changes with vital implications in normal and pathological cardiovascular conditions (96–98). EVs are classified into three subtypes, exosomes, microvesicles, and apoptotic bodies. While exosomes are continuously formed, microvesicles and apoptotic bodies require cellular activation or apoptosis, respectively (98). Exosomes are packaged during endocytosis and usually assembled with CD9, CD63, CD81, and components required for endosomal transportation. Such intraluminal vesicles are 50-100 nm in diameter and are released by exocytosis. MVs are generally larger, having diameters of 100-1,000 nm. The formation of MVs is initiated by the transfer of phosphatidylserine (PS) from the inner to the outer membrane leaflet. Release occurs through plasma membrane budding which results in membrane constituents mirroring the parental cell. Having diameters of 1-5 μm, apoptotic bodies are the largest EV type. These vesicles are formed during the final stage of apoptosis and also exhibit a PS-positive phenotype but show greater morphological diversity (99).

Under pathological conditions, EVs have been implicated in various atherogenic processes such as endothelial dysfunction (100), vascular inflammation (101, 102), and oxidative stress (103). Using wound-healing and cell-adhesion assays, circulating EVs from atherosclerotic patients were shown to enhance VSMC migration by +28.6% and adhesion by +15.9% compared to EVs from healthy patients. Further, *in vitro* foam cell-derived EVs (FC-EVs) were investigated. Foam cells were formed from J774a.1 cells cultured with oxLDL and subsequently, EVs were isolated from the culture media. VSMC incubation with these synthetic FC-EVs led to comparable outcomes with +44.6% and +18.6% increases for migration and adhesion, respectively. EVs from normal macrophages (NM-EVs) showed no difference from control cells. Interestingly, FC conditioned media deprived of EVs showed results similar to isolated FC-EVs. The strongest effect was observed with complete FC media containing soluble factors and FC-EVs (97). This may suggest an additive effect of FC-EVs and other soluble factors secreted by foam cells. However, since the purification process is not perfect, it is also conceivable that instead two distinct fractions of soluble factors were isolated that were responsible for the observed effects. Proteomic analysis of FC-EV content revealed factors involved in “actin cytoskeleton regulation” and “focal adhesion” pathways. Furthermore, VSMCs cultured with FC-EVs showed increased expression of ERK and Akt, two protein kinases that are known to be involved in cell migration and proliferation (97). Therefore, EVs released by macrophage-derived foam cells may enhance the intimal accumulation of VSMCs through ERK/Akt, while other soluble factors further stimulate VSMCs.

In a similar study, the differential analysis of EVs derived from oxLDL-stimulated human and mouse macrophages revealed enrichment of several miRNAs involved in macrophage motility and adhesion. miR-146a was among the most differentially represented miRNAs. Its transcription was significantly increased in atherosclerotic plaques from mice and humans, suggesting an essential role in both organisms. In the presence of oxLDL-stimulated macrophage EVs, macrophage migration toward CCL2 was significantly inhibited *in vitro*. Further, *in vivo* emigration of peritoneal macrophages in response to LPS injection was also impaired. EVs derived from knockout or siRNA-mediated knockdown of miR-146a increased the migration of naive macrophages compared to EVs from wt mice (96). EVs derived from oxLDL activated macrophages may modify motility and inhibit migration in naive macrophages. Thus, newly infiltrated macrophages may be immobilized and entrapped in atherosclerotic lesions.

Finally, beyond transfer of soluble factors or miRNAs, transwell co-culture experiments with fluorescently labeled cholesterol demonstrated the transfer of oxLDL and acLDL from macrophages to VSMCs. VSMCs formed lipid droplets in response to the co-culture conditions and switched their phenotypes. Interestingly, LDL uptake was much more modest in VSMCs cultures in the absence of macrophages, suggesting a transport mechanism between both cell types (104). That being said, in contrast to macrophage-derived foam cells, those originating from VSMCs generally exhibit reduced phagocytic and efferocytic activity, which may entail accelerated necrotic core growth and inflammation *in vivo* (4). After 14 days of co-culture, VSMCs showed enhanced phagocytic activity and expression of typical macrophage markers, including *Cd68* and *Mac2*. Xenogenic co-cultures (rat VSMCs and human macrophages) demonstrated that no human smooth muscle marker transcripts were expressed during transwell co-culture, but that cell-cell contact was instead required. Endosomal transportation was ruled out; transfection of macrophages with the reporter LAMP1-mKate2 revealed that macrophage-derived lysosomes were responsible for the transfer of LDL (104). Given that cell-contact was required for cholesterol uptake, close proximity to lipid-laden macrophages may be crucial for switching VSMCs to foam cells, especially during early atherosclerosis. Other independent pathways are likely given the large degree of redundancy observed in atherogenic processes.

### Communication Through Direct Contact

**Figure 3:**
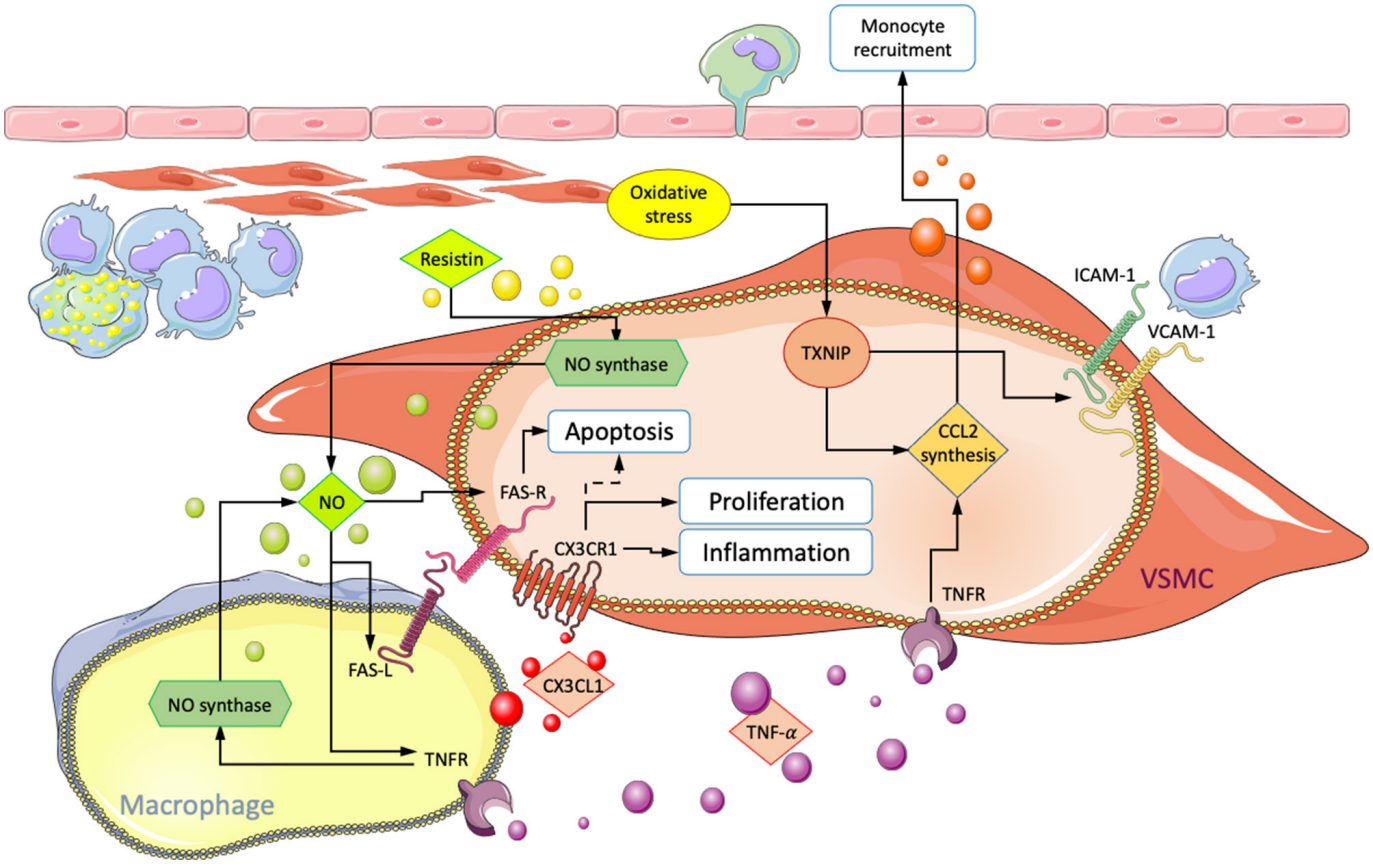
Summary of interactions involving physical contact between macrophages and VSMCs in atherosclerosis. Arrows point in the direction of enhanced processes, and dotted lines indicate inhibition. CX3CL1/CX3CR1 interaction leads to increased VSMC accumulation and inflammation. ROS-mediated increase in VCAM1/ICAM1 and CCL2 result in enhanced subendothelial macrophage retention and monocyte recruitment, respectively. Fas-mediated VSMC-apoptosis and the influence of NO, TNF-α, and resistin on FAS-R/L surface expression.

Communication between macrophages and VSMCs may also occur through direct contact between surface-expressed molecules such as ICAM-1, VCAM-1, and CX3CL1 with their corresponding receptors (Figure 3). Physical cell-cell interactions between both cell types have been implicated in various pro-atherogenic processes such as increased cytokine and metalloproteinase expression (71, 105), macrophage retention, and phenotypic modulation (106). CX3CL1 is a surface-bound chemokine that, together with its receptor CX3CR1, shows increased expression in atheroma resident VSMCs and macrophages. Binding through CX3CL1-CX3CR1 has been shown to upregulate the expression of inflammatory molecules in both cell types (29, 44, 71, 107). Conversely, disruption of the binding interaction significantly reduces the risk for atherosclerosis in humans and mice, suggesting an essential role (108, 109). Therefore, VSMCs and macrophages in physical contact may increase inflammation and promote atherogenesis. Also, CX3CL1 binding has been shown to favor VSMC proliferation and reduce apoptosis. Cellular binding may, therefore, also impact the number of VSMC-derived cells in atherosclerotic lesions. Since VSMCs can have both advantageous and detrimental effects, the impact of enhanced proliferation would likely depend on factors in the microenvironment that modulate VSMC phenotype (107).

Ablation of thioredoxin interacting protein (Txnip) in cultured VSMCs reduced NF-κB-mediated inflammation in response to oxidative stress. Expression of VCAM-1, ICAM-1, and production of CCL2 were significantly decreased. Macrophages from Txnip KO mice also showed lower CCL2 expression and a reduction of CX3CR1. For verification, assays were performed that showed a marked decrease of macrophage adhesion to VSMC in Txnip KO cells. Statistical significance was obtained from individual KOs, but the most substantial effect was obtained when Txnip was knocked out simultaneously in macrophages and VSMCs (25). This suggests a significant role of Txnip in the regulation of cellular interactions between macrophages and VSMCs during oxidative stress. However, validation from *in vivo* results is thus far missing. Additionally, Txnip has been demonstrated to be involved in NLRP3 activation, which may result in the modulation of VSMCs toward a macrophage-like phenotype with enhanced proliferation, migratory capabilities, and expression of inflammatory markers (25, 87).

Direct co-culture studies involving human cells also demonstrated macrophage induced VSMC apoptosis. Neutralization of Fas-L through antibodies and the use of NO inhibitors L-NAME and L-NMMA were shown to suppress cell death, suggesting that apoptosis is initiated *via* Fas ligand/receptor (Fas-L/Fas) interactions in a process that requires NO. NO may act by increasing the number of Fas receptors on VSCMs and Fas ligands on macrophages. Additionally, apoptosis was not observed in co-culture with monocytes, suggesting that macrophage maturation is required (110–112).

Further investigation showed similar results for TNF-α and its receptors TNF-R1 and -R2. At lower TNF-α concentrations (10 nmol/L), both Fas-L and TNF-α were required for VSMC apoptosis. Neither Fas-L nor TNF-α alone were sufficient to initiate cell death, suggesting a synergistic interaction. NO further amplified the process by increasing TNF-R1 expression in VSMCs, in addition to Fas. Antibody targeted neutralization of TNF-R1 and -R2 separately significantly reduced VSMC apoptosis in direct co-cultures with macrophages. Interestingly, selective TNF receptor neutralization on only macrophages also had an inhibitory influence. Macrophages with either of the two receptors neutralized exhibited reduced NO synthesis and abolished surface Fas-L expression. These findings suggest a synergistic mechanism involving TNF-α, Fas, and NO. TNF-α can directly induce apoptosis in VSMCs but also acts indirectly by increasing surface Fas-L and NO synthase activity in macrophages. NO, in turn, further sensitizes VSMCs toward macrophage-mediated apoptosis and TNF-α signaling by increasing both surface Fas and TNF-R1 (50). Additionally, incubation of VSMC cultures with TNF-α has been shown to induce production and release of the monocyte chemoattractant CCL2 (113). Exposure to NO and subsequent sensitization of VSMCs toward TNF-α could enhance macrophage recruitment and inflammation. Binding to VSMCs *via* VCAM-1 has also been shown to suppress macrophage apoptosis in culture and induce foam cell formation. *In vivo*, this could further amplify intimal inflammation (106).

Resistin, an inflammatory adipokine produced by macrophages, has been shown to have opposing influences on VSMCs. In indirect co-culture with macrophages, the addition of resistin enhanced VSMC proliferation *via* protein kinase C epsilon (PKCε). However, in culture conditions allowing for cell-cell interactions, resistin inhibited VSMC proliferation and enhanced apoptosis (114). It has previously been demonstrated that resistin activates NO synthesis *via* PKCε (115). Consequently, it is conceivable that resistin amplifies apoptosis through increased NO production and subsequent surface Fas-L/Fas upregulation on VSMCs.

### By-Products of VSMC Death

Apoptotic VSMCs synthesize and release factors that can further impact various intimal cells, including other VSMCs and macrophages. Expression of cytokines in response to Fas-induced apoptosis was evaluated in a recent study. IL-6 and GM-CSF were found to be increased through a p38 dependent mechanism when VSMCs were stimulated by a Fas receptor agonist. Besides being mediators of inflammation, IL-6 acts as a mitogen in VSMCs while GM-CSF has diverse roles, including inhibition of proliferation and upregulation of ECM synthesis (19). Both molecules are also involved in the differentiation of monocytes to macrophages (116). By applying the transcription inhibitor actinomycin (ActD) prior to the induction of apoptosis, it was found that IL-6 and GM-CSF were synthesized after the onset of apoptosis. Stau and α-Fas-induced cell death showed increased expression profiles only in cells that were not treated with ActD, suggesting that IL-6 and GM-CSF were produced during apoptosis and not released from previously established reserves. Furthermore, only cell populations undergoing cell death were found to exhibit enhanced IL-6 and GM-CSF expression. Consequently, VSMCs change their cytokine profile during apoptosis to communicate with local cells. In culture, VSMC death further exacerbated apoptosis in neighboring cells. Nevertheless, examinations in live animals led to contrary results. Selective induction of VSMC apoptosis was achieved using the diphtheria toxin receptor (DTR) and timed DT administrations. Targeted cell-death induced enhanced VSMC proliferation in both normal vessels and after ligation (19). Apoptosis might, therefore, work against further escalations of cell-death and establish a reparative phenotype in VSMCs during vascular injury. Previous studies using DTR for targeted apoptosis have shown that DT administration after ligation promoted vascular remodeling and VSMC proliferation. *In vitro* induction of apoptosis resulted in increased IL-6 expression, proliferation, migration, and collagen synthesis thus validating the *in vivo* results (117). These studies reveal responses to vascular injury of otherwise healthy vessel segments. The influence of synthetic VSMCs undergoing apoptosis within the atherosclerotic microenvironment or during chronic apoptosis was not addressed. Conflicting *in vitro* data and the complexity of the diseased microenvironment highlight the possibility of a markedly different response *in vivo* (19). A study contrasting DTR induced VSMC-death in healthy and atherosclerotic mice showed no inflammation, proliferation, or alteration of contractile markers in healthy vessels while plaque vulnerability was enhanced in diseased mice. Cap area and collagen content were reduced while percent necrotic core area was increased (39).

Finally, defective efferocytosis in atherosclerotic plaques may lead to apoptotic cells undergoing secondary necrosis. During this process, potent inflammation-promoting factors such as IL-1 and HMGB1 are released (118, 119). HMGB1 is also released by activated macrophages and acts as an autocrine stimulant of inflammation (119). Apoptotic VSMCs undergoing secondary necrosis were shown to be particularly inflammatory through the combined release of IL-1α and IL-1β rather than IL-1α alone (118). *In vivo*, IL-1α acts primarily in early lesion, while IL-1β promotes plaque growth at later stages (120). Further, media conditioned with IL-1α induced the production of CCL2 in VSMCs, which may lead to amplified recruitment of macrophages *in vivo* (118).

## Conclusion

The early atherosclerotic microenvironment enhances detachment, migration, and dedifferentiation of VSMC from the tunica media. During this process, contractile protein expression is either reduced or lost and replaced by other markers (48). Transcriptional modulation may eventually switch VSMCs into distinct phenotypes. Over the course of the disease, some become foam cells and aggravate the disease through inflammation and necrotic core formation. Others migrate to the inner sub-endothelial layer to synthesize matrix components that stabilize the plaque. VSMCs can also adopt an osteogenic-like phenotype leading to calcification and plaque vulnerability. Negative consequences on plaque stability appear to mostly depend on the topological structure of calcification (9, 29). VSMCs are phenotypically highly malleable in atherosclerosis and capable of influencing disease progression in profound ways. Knowledge of different cellular subtypes and their functions in varying atherosclerotic plaque environments is critical to advancing our understanding of the disease and the development of novel therapeutics.

Past studies have established diverse means of communication between VSMCs and macrophages. Interactions between both cell types were shown to significantly impact disease progression, as demonstrated by the interruption of relevant signal transducers such as CX3CR1. The consequences of macrophage-to-VSMC communication vary considerably among different models and cellular contexts. For example, studies have demonstrated VSMC foam cell induction from oxLDL while during vascular injury-induced remodeling, macrophages evoked a phenotype consistent with ECM-producing synthetic cells. Changes in the ratio of synthetic to foam cells may considerably impact plaque stability and disease progression. Therefore, understanding VSMC and macrophage subtypes and their relative functions in different atherogenic environments is pivotal for advancing our understanding of the disease and developing novel therapeutic approaches.

*In vitro* transdifferentiation of VSMCs was generally achieved without physical cell-contact, suggesting a significant role for soluble factors; either directly released into the media or packaged into EVs. The current literature suggests that physical contact to macrophages predominantly modulates VSMC apoptosis and inflammation. NO, and TNF-α act in synergy to sensitize cells toward apoptotic signaling. Binding *via* VCAM-1 may additionally enhance macrophage survival and foam cell formation, thus potentially amplifying interactions. Diminishing VSMC populations and increasing inflammatory cell accumulation should translate *in vivo* into decreasing plaque integrity. However, VCAM-1, NO, and TNF-α are critical factors that could also tilt disease progression into different directions depending on their relative concentrations. This ambiguity further highlights the need for a more comprehensive understanding of cellular and molecular functions in diverging disease environments.

The high complexity and variety of atherosclerotic plaques constitute significant hurdles for deriving meaningful data from studies. Animal models offer a high degree of exploratory freedom within the context of a live organism but are hampered by substantial cross-species and even intra-species differences. Genetic and dietary modifications are applied to align the animal pathophysiology more closely to that of humans but may themselves introduce confounding variables (121). Murine models have been most extensively used for studying atherosclerosis owing to the ease of genetic manipulations, low overall cost, and short breeding time frames. The current literature indicates significant overlap of relevant risk factors, genes, and pathways in atherosclerosis between mice and humans. However, important differences can also not be ignored (122). C-reactive protein (CRP), for example, has been established to be central to human inflammation and atherosclerosis but not to mice. Plaque instability and rupture are also not accurately represented by mouse models (123). Importantly, classically (M1) and alternatively (M2) activated macrophages are less clearly defined in humans (124). Differences in cellular functions may significantly alter the composition of atherosclerotic plaques and compromise translatability to humans.

Culture systems abstract from the complexity of animal models and allow focusing on specific aspects of cellular functions. However, findings are only meaningful if disease conditions can be reasonably replicated, which is challenging and has revealed inconsistencies. Cells in culture have undergone phenotypic transition and exhibit enhanced proliferation and susceptibility toward apoptosis which may explain some of the conflicting findings between *in vitro* models and live animals. Additionally, distinct molecular environments can radically influence cellular functions, as evident from the diverse modes of interaction between macrophages and VSMCs. Simple co-culture systems may lack potentially relevant signals from excluded cell types and ECM constituents. ECs, for example, continuously communicate with intimal cells and are often neglected. Multi-cell culture systems are useful if each cell type exhibits a phenotype reasonably consistent with what would be expected *in vivo*. However, the issue of distorted phenotypes may compound in cultures with multiple cells. Promising findings may result from novel models that mimic human disease conditions more accurately and permit higher throughput experimentation. New models could emerge from 3D cultures that mirror specific plaque environments including different cellular subtypes and ECM components.

## Data Availability Statement

The original contributions presented in the study are included in the article/supplementary material, further inquiries can be directed to the corresponding author/s.

## Author Contributions

JB-J and SL have been extensively involved in the preparation, writing, and editing of this review. MT contributed to the reviewing and editing, supervision, funding/resources, project administration and validation of the work. All authors contributed to the article and approved the submitted version.

## Funding

This work is supported by project grants to SL and MT from the Canadian Institutes of Health Research.

## Conflict of Interest

The authors declare that the research was conducted in the absence of any commercial or financial relationships that could be construed as a potential conflict of interest.

## Publisher's Note

All claims expressed in this article are solely those of the authors and do not necessarily represent those of their affiliated organizations, or those of the publisher, the editors and the reviewers. Any product that may be evaluated in this article, or claim that may be made by its manufacturer, is not guaranteed or endorsed by the publisher.
