# Corrigendum: Molecular interactions between vascular smooth muscle cells and macrophages in atherosclerosis

**DOI:** 10.3389/fcvm.2024.1462284

**Published:** 2024-08-12

**Authors:** Jahnic Beck-Joseph, Maryam Tabrizian, Stephanie Lehoux

**Affiliations:** ^1^ Biomat'X Research Laboratories, Department of Biomedical Engineering, McGill University, Montreal, QC, Canada; ^2^ Department of Medicine, Lady Davis Institute, McGill University, Montreal, QC, Canada

**Keywords:** atherosclerosis, coronary artery disease, inflammation, cell crosstalk, smooth muscle cells, monocytes/macrophages

A Corrigendum on Molecular interactions between vascular smooth muscle cells and macrophages in atherosclerosis By Beck-Joseph J, Tabrizian M and Lehoux S. (2021). Front. Cardiovasc. Med. 8:737934. doi: 10.3389/fcvm.2021.737934

In the published article, there was an error in the author list, and author Maryam Tabrizian was erroneously excluded. The corrected author list appears below.

Jahnic Beck-Joseph^1^, Maryam Tabrizian^1^ and Stephanie Lehoux^2^

As such the Author Contributions have also been updated. The corrected Author Contributions appear below.

JB-J and SL have been extensively involved in the preparation, writing, and editing of this review. MT contributed to the reviewing and editing, supervision, funding/resources, project administration and validation of the work. All authors contributed to the article and approved the submitted version.

The authors apologize for this error and state that this does not change the scientific conclusions of the article in any way. The original article has been updated.

In the published article, there was an error in the Funding statement. Maryam Tabrizian’s name was absent from the statement. The correct Funding statement appears below.

